# *BIRC5* Gene Disruption via CRISPR/Cas9n Platform Suppress Acute Myelocytic Leukemia Progression

**DOI:** 10.29252/ibj.23.6.369

**Published:** 2019-11

**Authors:** Manizheh Narimani, Mohammadreza Sharifi, Mohammad Saeed Hakhamaneshi, Daem Roshani, Mohammad Kazemi, Seyed Hossein Hejazi, Ali Jalili

**Affiliations:** 1 Cancer and Immunology Research Center, Kurdistan University of Medical Sciences, Sanandaj, Iran;; 2 Department of Genetics and Molecular Biology, School of Medicine, Isfahan University of Medical Sciences, Isfahan, Iran;; 3 Skin Diseases and Leishmaniasis Research Center, Department of Parasitology & Mycology, School of Medicine, Isfahan University of Medical Sciences, Isfahan, Iran

**Keywords:** Acute myelocytic leukemia, CRISPR, Gene editing, Survivin

## Abstract

**Background:**

Acute myelocytic leukemia (AML) is a clonal malignancy resulting from the accumulation of genetic abnormalities in the cells. Human baculoviral inhibitor of apoptosis repeat-containing 5 (BIRC5), encodes survivin, is one of only a handful of genes that is differentially over-expressed in numerous malignant diseases including AML.

**Methods:**

The *BIRC5 *was silenced permanently in two AML cell lines, HL‑60 and KG-1, via the CRISPR/Cas9n system. After transfection of CRISPR constructs, genomic DNA was extracted and amplified to assess mutation detection. To evaluate *BIRC5* gene expression, quantitative real-time PCR was performed. Also, MTT cell viability and Annexin‑V/propidium iodide flowcytometric staining were performed, and the data were analyzed using the Kolmogorov-Smirnov, Levene's, and ANOVA tests.

**Results:**

The results indicated that Cas9n and its sgRNAs successfully triggered site-specific cleavage and mutation in the *BIRC5* gene locus. Moreover, suppression of *BIRC5* resulted in the reduction of cell viability, and induction of apoptosis and necrosis in HL60 and KG1 suggested that the permanent suppression of *BIRC5* remarkably dropped the gene expression and cells viability.

**Conclusion:**

This study reinforces the idea that *BIRC5* disruption via Cas9n:sgRNAs has favorable effects on the AML clinical outcome. It thereby can be a promising candidate in a variety of leukemia treatments.

## INTRODUCTION

Hematological malignancies are a complex and divergent group of diseases caused by abnormality in hematopoietic cells and categorized into three types of leukemia, lymphoma, and myeloma^[^^1^^]^. Leukemia is generated from malignancies in the bone marrow^[^^2^^]^ and increased white blood cell count^[^^3^^]^. Acute myelocytic leukemia (AML) is a clonal malignancy resulting from the aggregation of immature myeloid precursors called blast, due to the occurrence of more than one driver genetic mutations^[^^4^^,^^5^^]^. AML accounts for more than 80% of leukemia among adults. The diagnosis average age is 66 years, and the treatment results are poor such that in the most cases lead to disease relapse^[^^6^^]^. Up to now, treatment modalities except for acute promyelocytic leukemia remain relatively constant due to disease complexities and diverse patho-physiologies^[^^7^^-^^9^^]^. Therefore, there is an urgent need to precisely dissect fundamental molecular mechanisms regulating leukemia and empower personalized medicine strategy via technological advanced tools^[^^10^^]^. In this regard, concurrent targeting multiple components of survival, proliferation, and apoptotic pathways in leukemic cells are all active areas of pre-clinical development and clinical trials to struggle towards the best treatment^[^^8^^]^. 

Profound expression of survivin in leukemic cells versus absence in normal differentiated adult cells highlight the biological importance of this oncoprotein in the biology of leukemia^[^^11^^-^^13^^]^. Beyond that, a strong correlation has been confirmed between highly survivin levels and cancer clinicopathological features^[^^14^^]^ as well as enhanced tumor drug resistance^[^^15^^]^ in numerous malignancies. Human baculoviral inhibitor of apoptosis (IAP) repeat-containing 5 (*BIRC5*), belongs to the IAP family, encodes survivin protein as a bifunctional regulator of apoptosis inhibition and cell cycle progression^[^^16^^,^^17^^]^. Survivin consists of only one structural feature of IAP molecules, designated as the BIR domain, a well-conserved N-terminal ∼70 amino acids that manages a zinc ion through cysteine and histidine residues^[^^18^^]^. The total length of protein-coding *BIRC5* sequences is about 11,440 bp on chromosome 17q25.3 and contains six exons (RefSeq classification), where yields five alternative splice variants, along with survivin wild type^[^^19^^,^^20^^]^, as illustrated in Table 1. It is noteworthy that overexpression of this protein results in the deactivation of tyrosine kinase receptors, such as receptor epidermal growth factor 1, 2ERBB, insulin-like growth factor-1(IGF-1), and its receptor (IGF-1R); besides, it triggers some cell survival signaling pathways, including PI3K/Akt, MEK/MAP, mTOR, STAT, and HIF-1^[^^21^^,^^22^^]^. Last but not least, taking into account the diverse facets of survivin expression in normal and neoplastic cells, it is nowadays capturing ample interest both as a prominent prognostic biomarker and as a potential cancer-targeted therapy^[^^23^^]^. In this regard, several pharmacological and genetic approaches have been used to interfere with survivin function, but none of them permanently silence it; hence, using CRISPR-Cas9 toolkit to knockout this eminent protein could pave the way for understanding the survivin precise mechanism. 

The magical multipurpose tool, CRISPR-Cas9, has opened a new window into the cancer etiology such that it can elucidate individual genes role in the tumor development. However, like any nascent technology, the CRISPR-based assay system has some potential pitfalls, including promiscuous off-target activity by Cas9. To address this issue, various preventative strategies have been employed, such as introducing purified Cas9 straight into target cells, using Cas9 nickase (Cas9n), decreasing sgRNA sequences by 2–3 nt, and exploiting additional two guanines at the 5ˊ terminus of gRNA immediately juxtaposed to the target-complementary region^[^^24^^-^^26^^]^. 

Having inactivated one of the Cas9 nuclease domains, HNH (H840A) or RuvC like (D10A), mutated Cas9n can just be able to induce a nick (single strand break) at target DNA instead of a cut (DSB [double-strand break]). As rule of thumb, upon creating two nicks in close proximity to other on DNA opposite strands, the cell machinery NHEJ pathway may consider it as a DSB and trigger to repair the desired target. With that in mind, the almighty CRISPR/Cas9n system utilizes a paired Cas9n associated with two sgRNAs to generate NHEJ-mediated gene knockout while diminishing drastically unwanted off-target^[^^27^^]^. On the clinical application side, even low off-target occurrence may cause an irreparable damage to gene therapy strategies because of unfavorable medical treatment outcomes. To date, astonishing achievements in the well-conceived CRISPR/Cas technology have been gained due to its cost-effectiveness, simplicity, specificity, and accuracy. However, taking additional great leaps toward being a stem of hope for personalized *therap*y, especially in the field of cancer treatments, are required. In the present study, we define that CRISPR-Cas9n vectors plus its sgRNAs could efficiently suppress *BIRC5* expression *in vitro*, which may enhance AML apoptosis and eventually improve o*utcomes in patients with* *leukemia*. We also verify and confirm that *BIRC5* oncogene is a promising new avenue in the treatment of cancers, such as leukemia. 

**Table 1:** Survivin protein-coding variants

**Transcripts**	**Exon count**	Transcript length** (bp)**	Translation length (aa)	Protein size (kDa)
Survivn wild type (isoform 1)	Four exons: 1, 2,3, and 4	2630	142	16.4
Survivin ΔEx3 (isoform 2)	Three exons: 1, 2, and 4	2446	137	15.6
Survivin 2B** (**isoform 3)	Five exons: 1, 2, 2B, 3, and 4	2711	165	18.6
Survivin 3B	Five exons:; 1, 2, 3, 3B and 4	492	121	12.5^[44]^
Survivin 2α	Two exons: 1, 2 + 32 nts from intron 2	568	74	8.5
Survivin 3α	Two exons: 1, 2 +197 nts of the 3’ end of intron 2	386	78	Not reported

## MATERIALS AND METHODS

**Cell culture**

This study included two different types of AML cell lines: human KG-1 cancer cell line, established from bone marrow cells of a patient with erythroleukemia evolving to acute myelogenous leukemia^[^^28^^]^, and HL-60 (human promyelocytic leukemia) cells, which were derived from a 36-year-old woman^[^^29^^]^. Both cell lines were obtained from Pasteur Institute of Iran, Tehran. To reach the exponential growth phase, the cell lines were suspended in RPMI 1640 medium (Gibco, USA) containing 10% heat inactivated (30 min, 56 °C) fetal bovine serum (FBS; Gibco), 1% Glutamax (Gibco), 1% penicillin/streptomycin (Sigma, USA) in fully humidified atmosphere at 5% CO_2_ and 37 °C until 70–80% confluence.

**Vector construction and expression**

CRISPR/Cas9n system was purchased from GeneCopoeia (Rockville, MD, USA). For CRISPR/Cas9n-based *BIRC5* knockout, Cas9n expression clone containing CBh promoter and nuclear localization signal sequences were used. sgRNAL and sgRNAR encompassed the U6 promoter, and their sequences were: 5ˊ CCAGGCAGGGGGCAACGTCG 3ˊ and 5ˊ GCATCTCTACATTCAAGAAC 3ˊ, respectively, which both of them recognized the target sequences located on the opposite strands of exon 1. Also, the negative control (scramble) vector for pCRISPR-SG01 was constructed as the same as sgRNA plasmid backbone, which did not contain a sgRNA sequence. All of the vectors were introduced by chemical transformation into the competent *E. coli* DH5α for cloning purposes using a selectable marker of ampicillin. These strains were cultured on Luria-Bertani (LB) agar plates supplemented with 1 mg/ml of ampicillin at 37 °C and subsequently, in LB broth, a liquid culture containing ampicillin in a 37 °C shaking incubator at 180 rpm/min speed. After that, all of the plasmids were purified with EndoFree Plasmid Maxi Kit (Qiagen, Germany) according to the manufacturer's protocol.

**Cell transfection**

In transient co-transfection for stable *BIRC5* knockout, 8 × 10^5^ cells were dispensed into each well of a six-well plastic tissue culture plate. Each well contained 2 ml of RPMI 1640 just prior to transfection. Cells were then transfected with the Cas9n and related sgRNAs, as well as scramble (negative control) using Lipofectamine 3000 (Invitrogen, USA). For a single well of a 6-well dish, 2500 ng of plasmid DNA, 5 µL of P3000 Reagent, 6 µL of Lipofectamine 3000 reagent, and 250 µL of Opti-MEM were used, according to the supplier's protocol. After six hours, supplemented media consisting of 10% FBS and 1% penicillin/streptomycin were added to each well. 

**DNA extraction and PCR amplification assay**

Genomic DNA (gDNA) was extracted using the PrimePrep^TM^ Genomic DNA Isolation Kit (GeNet Bio, Korea) 48 hours post transfection, and preliminary *quantification* of gDNA was performed by a NanoDrop spectrophotometer (WPA Biowave II, UK). The DNA region encompassing the CRISPR target site in *BIRC5* was amplified with Pfu High-Fidelity DNA Polymerase (Vivantis Technologies, Malaysia) using the sense 5'-GACTACAACTCCCGGCACAC-3' and antisense 5'-AAGGCATCAGGCATCTTACG-3' primers. PCR was performed under the following standard conditions: one-cycle initial denaturation of 3 min at 95 °C, followed by 35 cycles of 1 min at 95 °C, 1 min at 59 °C, and 1 min at 72 °C, with a final 10 min at 72 °C for post extension. Amplified PCR products were simply subjected to electrophoresis on 1.5% agarose gel prestained with DNA Green Viewer (Parstous, Iran) at 80 volts for 30-45 min. The 868-bp fragment was then excised from gel and purified using the AccuPrep™ Gel Purification Kit (Bioneer, Korea). 

**Surveyor mutation detection assay**

*BIRC5* oncogene knockout was assessed by the Surveyor Mutation Detection Kit (Integrated DNA technologies, USA) according to the producer’s instructions. In short, 400 ng of equal amounts of test (transfected) and reference (untransfected) purified PCR products were mixed in a microtube for DNA duplex formation, as well as the correspondence control group defined as just 400 ng of reference DNA in a separate tube. The subsequent procedure to promote heteroduplex formation was heating the PCR products and then cooling them slowly, which was performed on a Bio-Rad C1000 thermocycler as 15 distinct steps, including (1) 95 °C for 10 min, (2) 95 °C to 85 °C ramping at ‐2.0 °C/s, (3) 85 °C for 1 min, (4) 85 °C to 75 °C ramping at ‐0.3 °C/s; (5) 75 °C for 1 min, (6) 75 °C to 65 °C ramping at ‐0.3 °C/s, (7) 65 °C for 1 min, (8) 65 °C to 55 °C ramping at ‐0.3 °C/s, (9) 55 °C for 1 min, (10) 55 °C to 45 °C ramping at ‐0.3 °C/s, (11) 45 °C for 1 min, (12) 45 °C to 35 °C ramping at ‐0.3 °C/s, (13) 35 °C for 1 min, (14) 35 °C to 25 °C ramping at ‐0.3 °C/s, and (15) 25 °C for 1 min. After reannealing, samples were immediately kept on ice, and 1/10 total volume of each reaction mixture was added to 1.5 M of MgCl_2_. The next step was to treat the products with 1 µL of Surveyor Enhancer S and 1 µL of Surveyor Nuclease, followed by 60 min incubation at 42 °C. Lastly, stop solution was added to 1/10 volume of each product, and digestion products were clearly analyzed by 2% agarose gel. 

**RNA extraction and quantitative real-time (qRT)-PCR assay**

Total RNA was extracted from cells using the YTA Total RNA Purification Mini Kit (Yekta Tajhiz, Iran) 48 hours after transfection, according to the supplier’s protocol. Complementary DNA was synthesized from 2 µg of total RNA as a template by using random hexamer. Reverse transcription was performed with the help of the RevertAid™ First-Strand cDNA Synthesis (Thermo Fisher Scientific, Germany) at 42 °C for 60 min, followed by RevertAid Reverse Transcriptase at 5 °C for 70 min with. qRT-PCR assay was carried out with Real Q Plus 2X Master Mix Green high ROX™ Kit (Ampliqon, Denmark). PCR amplification was performed to produce an amplicon of 249 bp in 10 μL of final reaction volume with the SYBR Green detection method using the Step one plus Real-Time PCR System (Applied Biosystems, USA). The sequences of the primer sets were as BIRC5-F, 5'-CGCATCTCTACATTCAAG-3'; BIRC5-R, 5'-ATGT TCCTCTCTCGTGAT-3'; GAPDH-F, 5'-AAGCTCAT TTCCTGGTAT-3'; GAPDH-R, 5′-5'-CTTCCTCTTG TGCTCTTG -3'. The cycle conditions used for all genes were consisted of an initial step of 95 °C for 15 min, followed by 40 cycles of 95 °C for 30 s, 51.6 °C for 30 s, and 72 °C for 30 s with one cycle melt curve stage of 95 °C for 30 s, 60 °C for 1 min, and finally, 95 °C for 15 s. Relative quantification of gene expression was calculated using the comparative C_t_ method and the equation 2^−ΔΔCt^ after normalization the mRNA levels of target gene with an endogenous housekeeping gene, GAPDH. 

**Cell viability assay**

Cell viability was analyzed by the 3-(4,5-methylthiazol-2-yl)-2,5-diphenyl-tetrazolium bromide (MTT) viability assay. In breif, growing cells were seeded onto 96-well plates in triplicate at a density of 5 × 10^3^ cells per well, 48 hours after transfection of cells with CRISPR/Cas9n vectors. Next, 20 µL of the colorimetric MTT reagent (Sigma-Aldrich, USA) was directly added to the culture medium of each well with the final concentration of 1 mg/ml. After the microplate was incubated at 37 °C for 4 h, the medium was discarded completely, and 100 µL of dimethyl sulfoxide (Sigma-Aldrich) was added and shaked for 10 min. Cell viability was quantified by scanning with a microplate reader (Bio-Rad Model 680, USA) equipped with a 570-nm filter.

**Apoptosis assay**

Forty-eight hours after transfection, cells were harvested for flow cytometric analysis. The Annexin V fluorescein isothiocyanate- and propidium iodide (PI)-stained cells were carried out using the Annexin V FLUOS staining kit (Roche, Germany) as recommended by the manufacturer’s instructions. In brief, 5 × 10^5^ cells were pelleted by centrifugation at 500 g for 5 minutes (4 °C) before 50 μL of incubation buffer was added to each tube. Afterwards, cell double staining was performed by 1 μL of Annexin V and 1 μL of PI. Subsequently, the samples were incubated in the dark at room temperature for 20 min and then examined immediately using FACSCalibur flow cytometer (Becton Dickinson Biosciences, USA) after the addition of 300 µL of incubation buffer.

**Statistical analysis**

All biological experiments in literature were run in triplicate and at least two repetitions. Statistical analysis was done with SPSS 22 software (IBM, USA), where data was depicted as mean ± standard deviation (SD or σ), and *p* < 0.05 defined statistically significant. The Kolmogorov-Smirnov test was performed to determine whether data were normally distributed. Afterwards, homoscedasticity assumption was analyzed by Levene's test together with the one-way analysis of variance test (ANOVA), followed by the Scheffé's method for the analysis of variances. Moreover, flow cytometry data were analyzed using BD CellQuestPro software.

## RESULTS

**Design of the sgRNAs targeting human** ***BIRC5*** **gene**

**Fig. 1:**
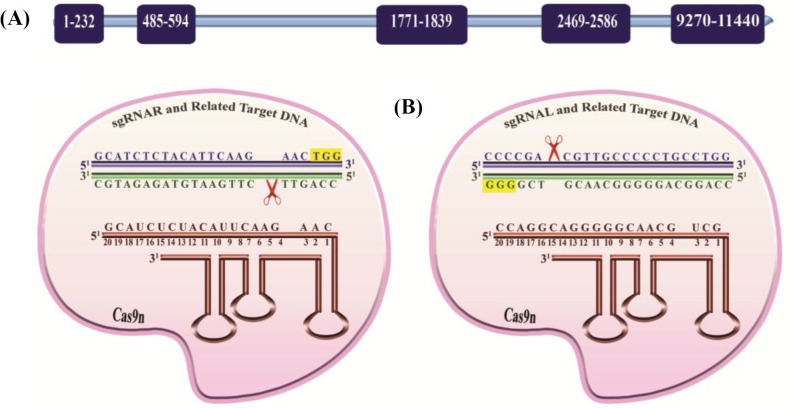
Illustration of the designed CRISPR/Cas9n system for *BIRC5* gene knockout. (A) Schematic diagram of *BIRC5* gene exons and their corresponding nucleotide positions. (B) Presentation of sgRNA sequences-directed CRISPR/Cas9n system and their corresponding regions in *BIRC5*. Protospacer adjacent motif sequences are labeled in yellow; sgRNAs in brown, as well as sense and anti-sense strands of the *BIRC5* gene are shown in navy blue and dark green, respectively

In the present study, we first *retrieved* the sequence of *BIRC5* (*NCBI* Reference Sequence (RefSeq): NG_029069.1) and then performed designation of sgRNAs. *BIRC5* gene is located on the telomeric position of chromosome 17q25.3 in forward strand, and 11 transcripts has been detected in this gene; six of them are transcribed to protein and three of which, *BIRC5-2B*, *BIRC5-WT*, and *BIRC5-deltaEx3*, are the most important. To knockout the gene of interest, we used the D10A nickase mutant of Cas9 (Cas9n) along with a dual sgRNA. Both sgRNAs are in principle composed of 20 nt in length, preceded by the canonical trinucleotide 5ˊ-NGG, the protospacer adjacent motif. In our study, sequences that contained this motif inside exon 1 of *BIRC5* were identified. Afterwards, to determine whether candidate sequences were unique in the genome or not, they were analyzed by BLAST. The salient features of DNA target sequences and their corresponding sgRNAs are detailed in Figure 1.

**Verification of the sgRNAs targeting human** ***BIRC5*** **gene** **effect**

**Fig. 2:**
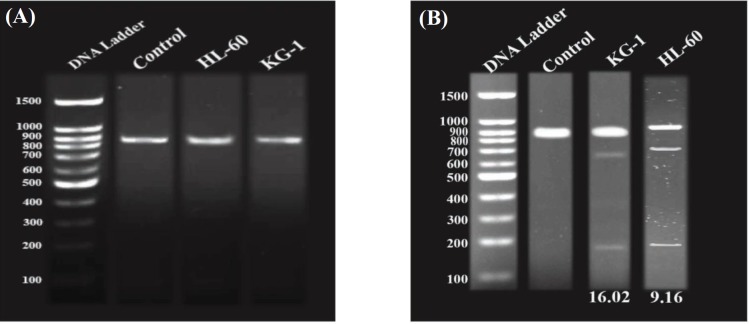
CRISPR/Cas9n-mediated cleavage at *BIRC5* locus in acute myelocytic leukemia cells. (A) PCR detection;(B) Surveyor assay of CRISPR/Cas9n activity in KG-1 and HL-60 cell lines. The numbers on the left represent the sizes of the DNA Ladder (bp). The numbers at the bottom of the gel indicate mutation percentages measured by band intensities

To characterize the functionality of Cas9n and their sgRNAs on the permanent disruption of *BIRC5* gene, we used Surveyor nuclease assay. This method is a mismatch-specific DNA endonuclease for indel mutations identification through the generation of DSBs and subsequent NHEJ DNA repair mechanism. Briefly, each of the sgRNAs and Cas9n cleaved one of the DNA strands, which were in turn mediated through the neighboring nicks detection as a DSB and led to NHEJ activation. Despite the off-target activity reduction of this system, the on-target efficiency typically tends to be unchanged. Also, the gDNA cleavage created breaks at 233 bp and 176 bp positions on the sense and anti-sense strands of the target DNA using sgRNAR and sgRNAL, respectively (Fig. 2). According to these results, we found that these sgRNAs successfully triggered site-specific cleavage in the *BIRC5* gene locus.

**Fig. 3:**
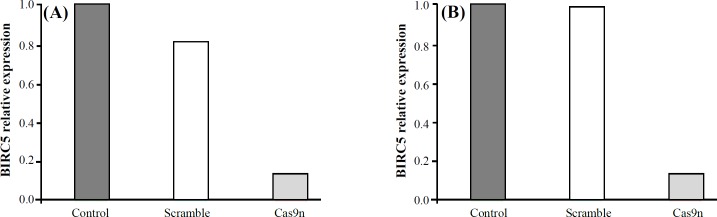
Quantitative evaluation of *BIRC5* expression in HL-60 (A) and KG-1 (B) cell lines via qRT-PCR 48 h after transfection. Relative expression values were normalized assigning the value of the cells in control groups to 1.0. Error bars represent mean ± SD of biological replicates from one experiment (*p* < 0.0001)

**Evaluation of gene expression in cells**

To confirm that *BIRC5* was thoroughly suppressed in AML cells, we surveyed the expression of *BIRC5* by quantitative real-time (qRT)-PCR. In Cas9n system, *BIRC5* was on the steady state levels of utmost expression in the control (untreated) and negative control (scramble) groups, whereas a corresponding severe reduction was observed in the treated groups in each iteration (Fig. 3). Having analyzed the data of qRT-PCR using ΔΔC_T_ method, the results were according to expectations, which mean that spectacular changes in gene expression were statistically observed between scrambles (negative control) and treated groups with sgRNAs accompanied by Cas9n. 

**Verification of cell viability**

**Fig. 4:**
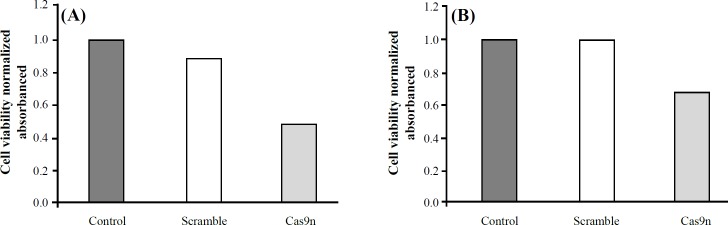
Proliferation of the various groups-transfected HL-60 (A) and KG-1 (B) cells quantified using a MTT assay 48 h after transfection. The viability of the untreated cells was considered as 100%, and the viability of other groups is presented as the percentage of the untreated cells. Data were mean ± SD of three independent experiments (*p* < 0.0001)

We attempted to define the basis for the inevitable effects of knockout *BIRC5* using Cas9n system on cell viability. To this end, we used the colorimetric MTT assay to monitor only metabolically active cells; therefore, the yellow tetrazole salt reduced to purple insoluble formazan product by oxidoreductase enzymes reflected the number of viable cells present. As illustrated in Figure 4, evaluation of MTT results suggested that the mean of living cells in the control and negative control (scramble) groups was approximately identical; however, treated groups with sgRNA/Cas9n demonstrated drastically a decrease in living cells. 

**The effect of CRISPR/Cas9n technology in suppressing** **cell growth**

**Fig. 5:**
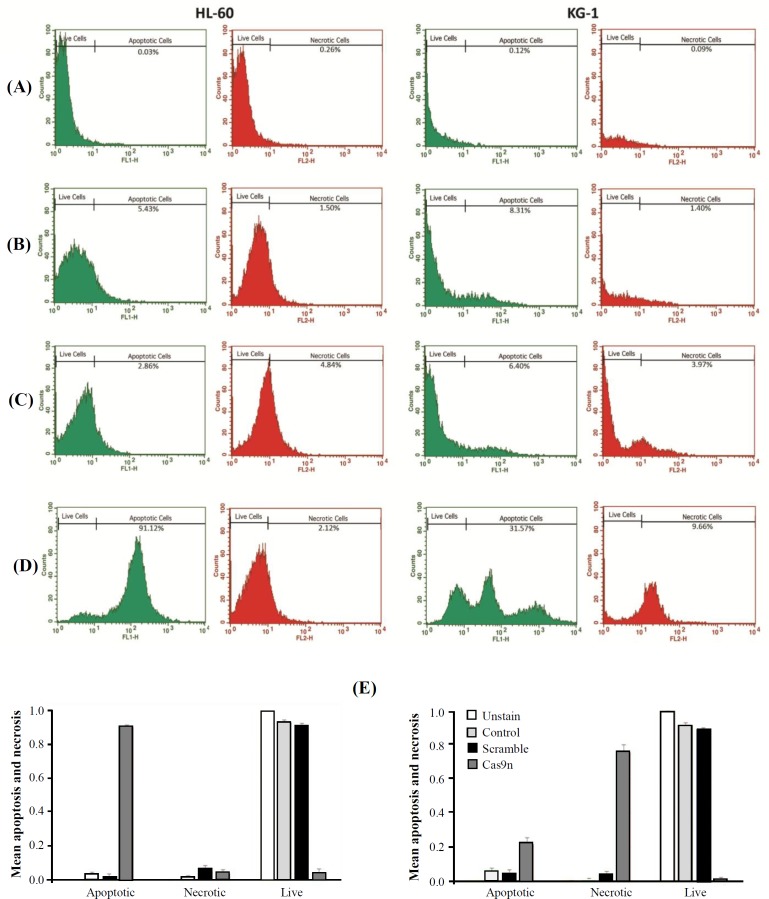
Targeting of survivin resulted in the induction of apoptosis in leukemic cell lines. Significantly enhanced apoptosis was monitored by flow cytometry in the treated versus untreated groups in HL-60 and KG-1 cell lines 48 h after transfection. (A) unstained, (B) control, (C), scramble, and (D) Cas9n. Representative cytofluorometric graphs are shown in (E) for HL-60 and KG-1 cell lines

To determine whether *BIRC5* suppression by the CRISPR/Cas9n editing system is sufficient to induce a change in cell fate, we applied this system to the leukemia cell lines, KG-1 and HL-60. Two days after transfection, we measured substantial apoptosis and necrosis in the cells transfected with CRISPR/Cas9n by flow cytometry. As a whole, cell surface phosphatidylserine is mainly restricted to the plasma membrane inner leaflet. A classic feature of early apoptotic cells is the translocation of phospha-tidylserine to the plasma membrane outer layer. This phenomenon can be detected by Annexin V protein, which labeled with fluorochrome, fluorescein isothiocyanate. Having progressed cell membrane destruction, a fluorescent molecule PI can be used to capably differentiate between apoptotic and necrotic cells. PI was generally excluded from viable cells because of membrane impermeant and bound to DNA by intercalating between the bases. As illustrated in Figure 5, the most significant apoptotic cells were observed in treated sgRNA/Cas9n groups. However, there was a little change between the percentage of apoptotic cells in the control and scramble groups, and both had statistically noticeable differences with the *BIRC5* sgRNA/Cas9n group. Likewise, the number of necrotic cells for sgRNA/Cas9n groups was statistically significant (*p *< 0.0001). Taken together, silencing *BIRC5* expression by CRISPR/Cas9n may hold clinical therapeutic promise to rescue acute myeloid leukemia by suppressing uncontrolled growth of these cells.

## DISCUSSION

The CRISPR/Cas9n research enterprise has been peculiarly zoomed in basic and clinical applications. In other words, in this day and age, CRISPR groundbreaking technology has *gathered a lot of attention to prevailing long-term challenge on gene editing. Here*, *w*e report the first successful use of Cas9n-mediated genome editing system to introduce permanent indel mutations into the human *BIRC5* oncogene in AML cell lines using a lipid-based transfection methodology. We aimed to explore the enormous potential of this system in generating knockout in human leukemia cells in culture and to elucidate the *BIRC5* mutation impact on cellular function.

A previous study has clarified that *BIRC5* gene is overexpressed in numerous malignancies such as leukemia and correlated with tumor progression and drug resistance^[^^11^^]^. The most distinctive feature of survivin is involvement in cell death regulation. This nodal protein cannot directly interact with^[^^30^^]^ caspases; it associates with X-linked IAP to repress caspase-dependent apoptotic pathway. Owing to its pivotal role in anti-apoptotic effects as well as promotion of cancer cell invasion and migration, survivin can be considered as a noticeable prognostic and metastatic factor in cancerous cells^[^^31^^]^. Several lines of evidence made it apparent that high *BIRC5* expression is associated with substantially inferior, poor, and adverse clinical outcome in AML^[^^12^^,^^13^^,^^30^^,^^31^^]^.

Investigators have previously established survivin gene isoforms overexpression (survivin, -2B, -Ex3, and -3B, except survivin-2α) in the human AML-M3 cell line, NB4, as well as patient’s bone marrow samples, wherein their expression declined after arsenic trioxide treatment as a front-line therapy^[^^12^^]^. In 2017, Pazhang *et al*.^[^^13^^]^ have demonstrated survivin down‑regulation in HL-60 cancer cells using embelin and celastrol, two anti-tumor agents, led to considerable *response improvement to chemotherapeutic* agents and diminished NF-κB activity, because of the crosstalk existence between X-linked IAP and NF-κB pathway. Another study has revealed that pretreatment with survivin siRNA could synergistically sensitize U-937 AML cells to anticancer drugs and could enable them to induce apoptosis^[^^11^^]^. To overcome uncontrolled cell growth and proliferation, we, therefore, hypothesized that *BIRC5* knockout with the *currently best* known CRISPR/Cas toolkit *would* confer cancerous cells depletion due to apoptosis augmentation in KG1 and HL-60 cells.

Given the extensive interest on gene editing tools, CRISPR is of paramount importance to change considerably face and pace of knockout in genes, which enables new specific target detection for various cancers including leukemia. Analysis of Cas9 nuclease, wild-type Cas9, has been illustrated as highly frequent off-target activity alongside robustly efficient on-target mutagenesis^[^^33^^,^^34^^]^. Thus, to diminish drastically Cas9 nuclease-mediated off-target with equivalently indel frequencies, an alternative strategy, Cas9n, comes into the picture. In spite of Cas9 wild type, Cas9n approach stands in need of two heminuclease domains to create a DSB at the target site^[^^33^^,^^35^^]^. In this investigation, to mediate *BIRC5* gene manipulation, we used Cas9n and two related sgRNAs. More interestingly, we envisioned that Cas9n strategy was most potential to generate genetically modified human cells, as well as minimally, if any, changes between Cas9 and Cas9n were observed. Indeed, the main reason for nearly similar effectiveness is nuclear localization signal incorporation into the Cas9n vector, which enhances cell permeability, eventually resulting in the high Cas9n system reproducibility for utmost precision genome editing. Consistent with our results, seminal studies have shown that the utility of applicable CRISPR-assisted NHEJ is profoundly effective in mammalian gene ablation^[^^36^^-^^39^^]^. Furthermore, we delineated an inverse correlation between survivin protein levels and apoptosis in AML cells. Having knocked out cells with CRISPR constructs, we detected an alarmingly increasing number of programmed cell death, confirming the survivin invaluable function in cancer development, cell survival, and proliferation. Our data are in close agreement with previous ones that unraveled survivin/*BIRC5* mechanistic events is the pivotal and ubiquitous nature of tumor progression and clinical manifestation; consequently, it could emerge as an attractive therapeutic target in cancer treatment^ [^^40^^-^^43^^]^.

Collectively, this study strengthens the idea that cationic lipid-delivered Cas9n:sgRNA is a feasible method for targeting *BIRC5* gene. More importantly, targeting *BIRC5* by CRISPR/Cas9n led to the induction of apoptosis and reduction of leukemic cells growth, implying that CRISPR/Cas9n is a suitable approach for therapeutic interventions for over-expressing tumors.
